# A family harboring an MLKL loss of function variant implicates impaired necroptosis in diabetes

**DOI:** 10.1038/s41419-021-03636-5

**Published:** 2021-04-01

**Authors:** Joanne M. Hildebrand, Bernice Lo, Sara Tomei, Valentina Mattei, Samuel N. Young, Cheree Fitzgibbon, James M. Murphy, Abeer Fadda

**Affiliations:** 1 The Walter and Eliza Hall Institute of Medical Research, Parkville, VIC 3052 Australia; 2 Department of Medical Biology, University of Melbourne, Parkville, VIC 3050 Australia; 3 Research Department, Sidra Medicine, Doha, 26999 Qatar

**Keywords:** Necroptosis, Diabetes

## Abstract

Maturity-onset diabetes of the young, MODY, is an autosomal dominant disease with incomplete penetrance. In a family with multiple generations of diabetes and several early onset diabetic siblings, we found the previously reported P33T *PDX1* damaging mutation. Interestingly, this substitution was also present in a healthy sibling. In contrast, a second very rare heterozygous damaging mutation in the necroptosis terminal effector, MLKL, was found exclusively in the diabetic family members. Aberrant cell death by necroptosis is a cause of inflammatory diseases and has been widely implicated in human pathologies, but has not yet been attributed functions in diabetes. Here, we report that the MLKL substitution observed in diabetic patients, G316D, results in diminished phosphorylation by its upstream activator, the RIPK3 kinase, and no capacity to reconstitute necroptosis in two distinct *MLKL*^*−/−*^ human cell lines. This MLKL mutation may act as a modifier to the P33T *PDX1* mutation, and points to a potential role of impairment of necroptosis in diabetes. Our findings highlight the importance of family studies in unraveling MODY’s incomplete penetrance, and provide further support for the involvement of dysregulated necroptosis in human disease.

## Introduction

Monogenic diabetes constitutes less than 5% of diabetes cases, of which the two main forms affect either newborns (NDM) or young adults (MODY)^1^. To date, there are 14 types of MODY named after the genes involved in each. MODY is inherited in an autosomal dominant mode, and despite being described as monogenic, it is not fully penetrant^2^. Families affected with the insulin promoter factor-1 (PDX1) MODY (or MODY4), for example, frequently do not segregate strictly with pathogenic PDX1 mutations, with unaffected carriers being common and some diabetic members lacking the mutations^3–7^. PDX1 is a transcription factor that regulates expression of key pancreatic genes, including those encoding insulin, somatostatin, glucokinase, islet amyloid polypeptide and glucose transporter type 2 genes^8^. Biallelic damaging mutations have been well characterized with severe consequences in humans and mice, involving pancreatic agenesis, NDM and death^9,10^. However, mice bearing a single allele knockout exhibit only a mild phenotype and do not develop diabetes^11^. Despite many of the reported MODY4 mutations studied in vitro and in vivo confirming a role in pathogenesis^3,4,6,7^, these studies stop short of explaining the phenomenon of incomplete penetrance. Additional environmental or genetic factors are clearly acting alongside PDX1 mutations in the etiology of MODY. In support of this idea, the selective, inducible inhibition of IKK/NF-κB signaling in pancreatic β-cells could induce bona fide diabetes in pre-diabetic adult *Pdx1*^*+/*−^ mice^12^.

Family studies have greatly contributed to the identification of pathogenic mutations and continue to yield important new insights even in the era of large cohort sequencing. While genome-wide association (GWAS) studies succeeded in revealing many disease-associated variants with small effects on phenotypic expression, rare variants with big or moderate phenotypic effects are overlooked. Here, we present findings gleaned from a family with five siblings, four of whom have diabetes in addition to their mother. We observed the previously described P33T PDX1 haploinsufficiency mutation in all patients and the unaffected sibling. This mutation has been previously reported in MODY patients, and functional analyses show reduced binding of the mutated PDX1 to the insulin promoter and reduced transcriptional activation in vitro^4^. Several in silico prediction tools confirmed a damaging effect of this mutation, while the high conservation score (PhyloP *p* value = 2.78E–06) indicates the functional importance of this substitution to protein function. However, relative abundance of the variant in the population, (global MAF of 0.0023, or 0.23% of all *PDX1* alleles genotyped in gnomAD^13^) is atypical of a mutation responsible for monogenic disease. The lack of strict allele-disease segregation (or penetrance) within this and other families also contradicts its classification as a Mendelian pathogenic gene variant. Therefore, it is classified as a variant of uncertain significance according to the American College of Medical Genetics and Genomics (ACMG) guidelines in ClinVar^14^. Through direct genomic comparisons, the presence of a healthy carrier in the family provides a great opportunity for finding modifier mutations that would explain the incomplete penetrance. Further sequence analysis revealed a rare mono-allelic damaging mutation in the Mixed Lineage Kinase Domain-Like (*MLKL*) gene, exclusively in the diabetic family members.

MLKL is the terminal executioner protein in the necroptosis cell death pathway^15–18^. Necroptosis is a lytic cell death pathway that is thought to have evolved as an arm of the innate immune response to pathogens^19–24^. While it is dispensable for mouse and human development^16,25,26^, dysregulated necroptosis has been widely implicated in infectious and non-infectious disease alike^19,26–32^. Necroptotic signals can emanate from several forms of extracellular and intracellular stimuli, but almost universally culminate in the formation of the ‘necrosome’. Within the necrosome, RIPK3 activity is enhanced by autophosphorylation^17,33–35^, which provides the cue for MLKL recruitment and phosphorylation. Upon phosphorylation of its regulatory pseudokinase domain, MLKL undergoes conformational switching to an activated form^16,36–41^, which is oligomerized and trafficked to the plasma membrane where it accumulates into hotspots that permeabilize the cell to cause lytic death^42–45^.

To date, relatively few human diseases have been associated with *MLKL* variant sequences, with substitutions so far genomically-linked to neurodegenerative spectrum disorder^26^, Alzheimer’s disease^31^, and Chronic Recurrent Osteomyelitis (CRMO)^27^. Here, we report a missense mutation in *MLKL* that segregates exclusively with affected members of a diabetic family. The amino acid substitution, G316D, fully ablates MLKL killing activity in human cells in vitro. These data suggest that impaired necroptosis may contribute to the penetrance of P33T PDX1 haploinsufficiency-induced diabetes in these patients.

## Results

### Case presentation

**Fig. 1:**
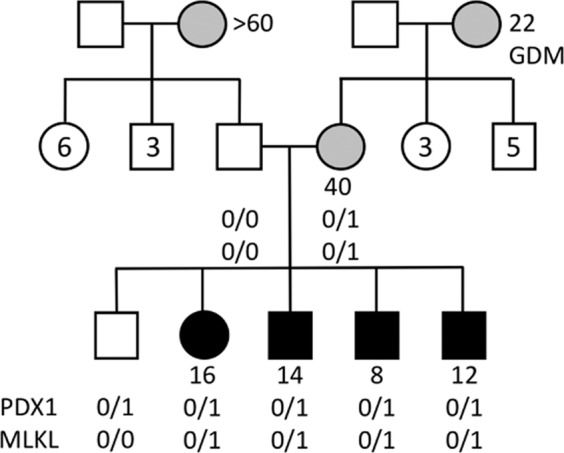
Pedigree of a non-consanguineous Palestinian diabetic family. Gray- and black-filled shapes indicate mild and severe diabetes, respectively. White-filled shapes represent the unaffected family members; numbers inside shapes reflect the number of unaffected siblings. Circles represent females while squares represent males. Numbers stated beside circles and squares refer to age of disease onset. GDM is Gestational Diabetes Mellitus. Genotypes listed are for PDX1 and MLKL mutations respectively.

A non-consanguineous family of Palestinian ethnicity has multiple generations and multiple siblings affected with diabetes (Fig. 1). Four out of five siblings have developed insulin dependent diabetes during their teens. Their mother developed mild diabetes at 40 years of age which she manages with diet, while her mother developed gestational diabetes that did not resolve. Within the nuclear family, all of the affected are lean to normal weight; the paternal grandmother had type 2 diabetes. We tested non-fasting glycosylated hemoglobin (HbA1c), endogenous insulin (c-peptide), and two autoantibodies, anti-glutamic acid decarboxylase (anti-GAD), and anti-thyroid peroxidase (anti-TP) antibodies (Table 1). All affected siblings had very low levels of endogenous insulin, and 3 were positive for at least one autoantibody. The mother showed a slight increase in c-peptide suggestive of insulin resistance. The older sibling remains unaffected at 31 years of age, the time of writing the manuscript.

**Table 1:** Summary of clinical measurements.

	Age	Sex	BMI	Disease duration	Treatment	HbA1c % (mmol/mol)^a^	c-peptide (ng/ml)^b^	anti-GAD	anti-TP
Mother	40	F	25	<1	Diet	7.4 (57)	5.37	na	na
Father	50	M	21	–	_	na	na	na	na
Unaffected sibling	27	M	23	–	_	6.1 (43)	2.63	na	na
Sibling 2	25	F	24	9	Insulin	8.8 (73)	0.05	Negative	Positive
Sibling 3	20	M	22	6	Insulin	9.1 (76)	0.01	Positive	Negative
Sibling 4	19	M	18	11	Insulin	10.3 (89)	0.01	Negative	Negative
Sibling 5	17	M	16	5	Insulin	12.7 (115)	0.65	Positive	Negative

^a^Normal range for HbA1c is 4.0–6.0%.

^b^Normal range for c-peptide 0.8–4.0 ng/ml.

### Oligogenic inheritance

**Fig. 2:**
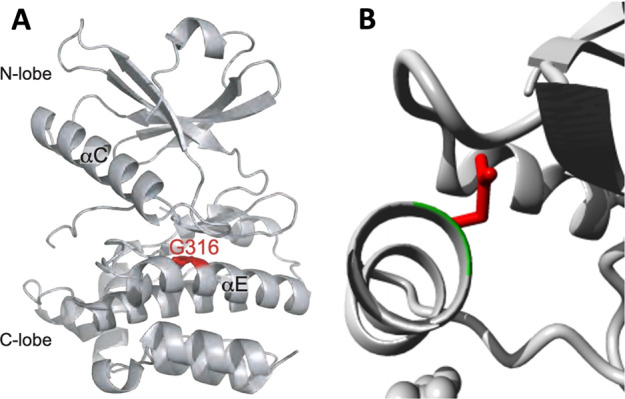
MLKL G316D mutation is predicted to affect the pseudokinase domain structure (PDB accession 4MWI)^38^. (**A**) The substitution is in the αE helix within the C-lobe. (**B**) A close up of the amino acid change showing the wild type residue in green and the mutant aspartic acid side chain in red. Figures drawn with Pymol (Schrodinger, LLC. 2010) and HOPE^58^.

Whole genome sequencing was performed for all members of the nuclear family. We examined CNVs (copy number variations), indels (insertions/deletions) and SNPs (single nucleotide polymorphisms), and applied strict variant filtering criteria such as a minimum read depth of 20, global allele frequency <1%, and prediction of pathogenicity by both SIFT and PolyPhen (see details in the methods). Variant analysis revealed the presence of a maternally inherited heterozygous mutation in *PDX1* c.97 C > A, p.P33T, (rs192902098) in all 5 siblings, including the healthy unaffected sibling (Fig. 1). Looking for potential modifier mutations, we extracted all genetic variants that were shared by the diabetic siblings, but absent in the unaffected sibling. Applying the same filtering criteria as above, we arrived at four substitution mutations listed in Table 2. One of these substitution mutations was in the gene encoding the Mixed Lineage Kinase Domain Like protein (MLKL G316D, rs375490660). The MLKL G316D substitution occurs within the αE helix within the C-lobe of MLKL’s C-terminal pseudokinase domain. The mutation is very rare (0.001% of MLKL alleles) and is not found in the homozygous state in the gnomAD database^13^. Owing to the steric bulk of an Asp relative to the native Gly (Fig. 2), and the accompanying introduction of a negatively charged sidechain, we predicted the G316D substitution would be deleterious to protein function.

**Table 2:** List of predicted pathogenic mutations found exclusively in diabetic siblings. M and P denote maternal and paternal inheritance, respectively.

Gene	GO annotation	Nucleotide change	Protein change	Genomic location (GRCh37)	snpID	MAF%	SIFT prediction	Genotype	Donor parent
MLKL	Necroptosis	c.947 G > A	p.Gly316Asp	16:74,716,558	rs375490660	0.001	Deleterious	Het	M
ERN2	Intrinsic apoptotic signaling pathway in response to endoplasmic reticulum stress	c.2831 C > G	p.Ala944Gly	16:23,702,246	rs56129167	0.53	Deleterious	Het	P
NIPAL4	Magnesium ion transmembrane transporter activity	c.361 G > A	p.Gly121Ser	5:156,890,239	rs370726117	0.003	Deleterious	Het	M
SPTBN4	Actin binding	c.2983 G > C	p.Glu995Gln	19:41,025,387	rs750181241	0.0008	Deleterious	Het	M

### G316D substitution impairs MLKL activation

**Fig. 3:**
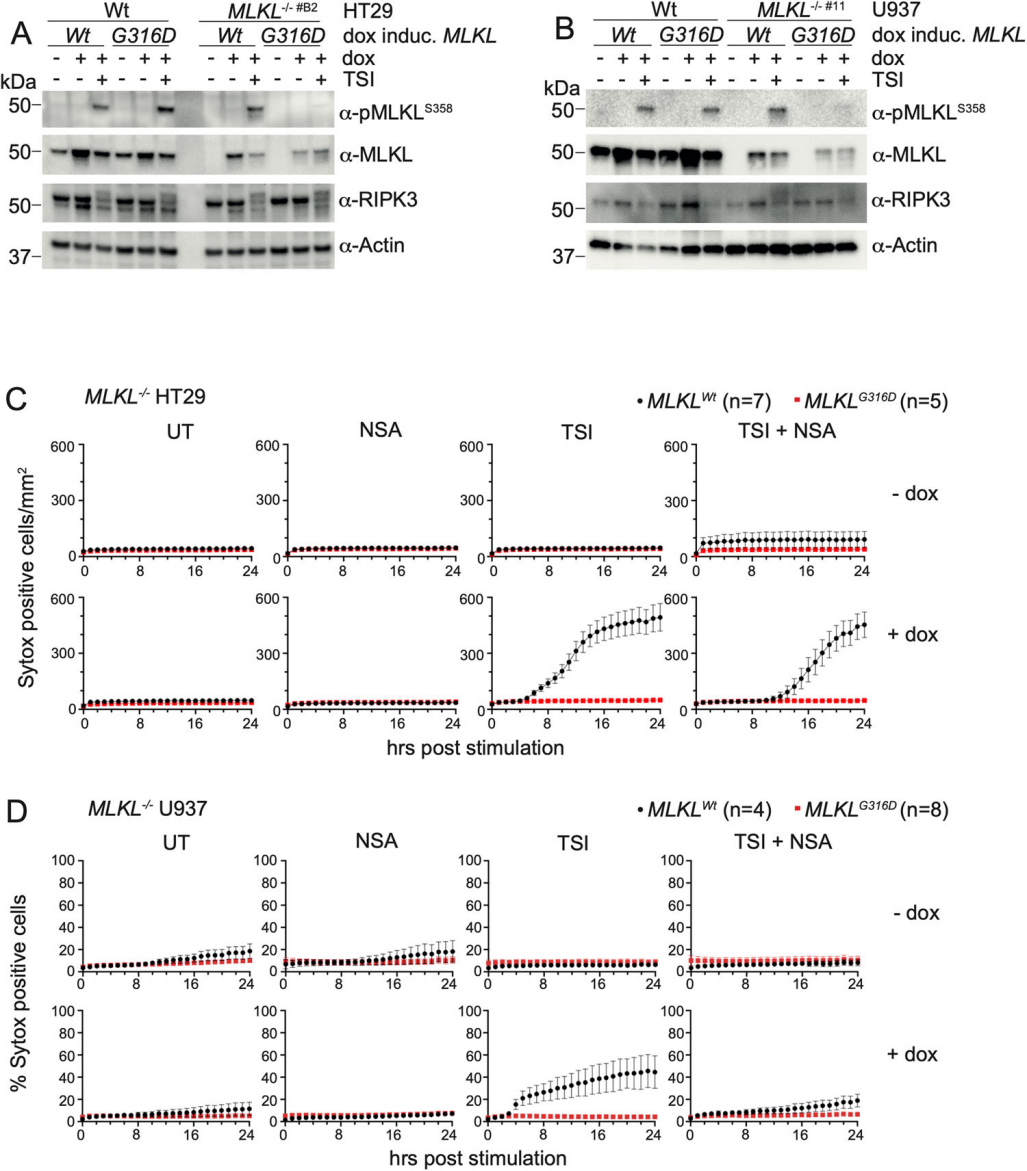
MLKL^G316D^ shows much reduced levels of Ser358 phosphorylation and no capacity to induce necroptosis in HT29 and U937 cell lines. (**A**) HT29s and (**B**) U937s were stably transduced with doxycycline (dox) inducible human *MLKL*^*WT*^ or *MLKL*^*G316D*^ expression constructs. MLKL and RIPK3 protein levels were analysed by Western blot after 5 h doxycycline in the absence or presence of a necroptotic stimulus (TSI). Images in (**A**) and (**B**) are representative of at least 3 independent experiments. *MLKL*^*−/−*^ HT29s (**C**) and U937s (**D**) expressing doxycycline inducible *MLKL*^*WT*^ (black circle) or *MLKL*^*G316D*^ (red square) were stimulated as indicated. Total number Sytox green positive cells per mm^2^ or proportion of Sytox green positive cells per mm^2^ were quantified over time using an IncuCyte automated cell imager. Plotted as mean ± SEM of at least 4 independent experiments, specific “n” as indicated. Necroptotic stimulus, TSI; TNF, Smac mimetic and IDN6556. Necroptosis inhibitor, NSA; necrosulfonamide.

To examine if the non-conservative Gly316Asp amino acid replacement altered the stability or function of MLKL in human cells, we examined this mutant relative to wild-type MLKL in two cell lines commonly used to study necroptotic signal transduction: the HT29 colon adenocarcinoma and U937 monocytic cell lines. *MLKL*^*+/+*^ and *MLKL*^*−/−*^ lines were stably-transduced with a doxycycline-inducible *MLKL*^*WT*^ or *MLKL*^*G316D*^ gene construct. All cell lines used showed similar levels of RIPK3 and equivalent levels of RIPK3 activation following the addition of the necroptotic stimulus TNF, Smac-mimetic and IDN6556 (TSI) as judged by upshift of the RIPK3 band on western blot indicating protein modification (Fig. 3a). Following a 5 h incubation of *MLKL*^*−/−*^ HT29 cells with 100 ng/mL doxycycline, exogenously encoded MLKL^G316D^ protein levels were ~50% that of exogenously encoded MLKL^WT^ in cells not stimulated by TSI (Fig. 3a, Supplementary Fig. 1a). Despite similar levels of total MLKL^WT^ and MLKL^G316D^ species detectable in *MLKL*^*−/−*^ HT29s stimulated with both doxycycline and TSI, phosphorylated MLKL (pS358) was virtually undetectable for G316D MLKL (Fig. 3a, quantified in Supplementary Fig. 1a). This reduced level of total MLKL expression and only trace levels of phosphorylated MLKL is also seen when G316D MLKL was expressed under the same conditions in *MLKL*^*−/−*^ U937 monocytic cell lines (Fig. 1b, quantified in Supplementary Fig. 1b), and at supraphysiological levels of exogenous MLKL^G316D^ expression (Supplementary Fig. 1c).

### MLKL^G316D^ compromises necroptotic effector activity

We next examined if MLKL^G316D^ could function as an effector of necroptotic cell death in cultured cell lines. Following the addition of 100 ng/mL doxycycline, which facilitates expression at almost physiological levels (relative to WT cells), MLKL^G316D^ failed to reconstitute any sensitivity to the necroptotic stimulus, TSI. This was the case in three independent *MLKL*^*−/−*^ clones of each of the HT29 (Fig. 3c) and U937 (Fig. 3d) cell lines. This was not due to any deficiency in upstream signaling, given the equivalent activation of RIPK3 apparent by western blot post TSI stimulation (Fig. 3a, b, Supplementary Fig. 1c) and equivalent activation of the extrinsic apoptotic pathway following stimulation with TNF and Smac mimetic in the absence of caspase inhibition (TS) (Supplementary Fig. 2a-HT29 and b-U937). Even when expressed at supraphysiological levels following the addition of 500 ng/mL doxycycline, MLKL^G316D^ failed to reconstitute any sensitivity to the necroptotic stimulus, TSI, in both *MLKL*^*−/−*^ HT29 (Supplementary Fig. 2c) and *MLKL*^*−/−*^ U937 (Supplementary Fig. 2d) cell lines. When expressed in an *MLKL*^*+/+*^ background at near physiological and supraphysiological levels, exogenous *MLKL*^*G316D*^ does not alter the maximal necroptotic cell death facilitated by endogenous wild-type MLKL in HT29s (Supplementay Fig. 3a). In *MLKL*^*+/+*^ U937s, the exogenous expression of *MLKL*^*G316D*^ reduces the maximal necroptotic cell death facilitated by endogenous wild-type MLKL up to 50%, but the same reduction is similarly observed for exogenous *MLKL*^*WT*^ under these conditions, precluding any conclusion about a specific dominant-negative effect of *MLKL*^*G316D*^ (Supplementary Fig. 3a, b).

## Discussion

While diabetes is a complex disease, monogenic forms of diabetes have been relatively easier to study and delineate the causative mutation. However, in many cases the pathophysiology remains enigmatic, including in MODY, where penetrance is incomplete and the mutations do not segregate fully with disease status. In this study we have taken advantage of classic Mendelian genetics in a family with multiple affected members, to discover rare variants that may interact to cause diabetes. In addition to the previously reported P33T *PDX1* mutation with incomplete penetrance, we found a very rare mutation in the key necroptosis protein, MLKL, present in the affected individuals only. Necroptosis proteins have been linked to glucose homeostasis in mice. MLKL, RIPK1 and RIPK3 were found to be upregulated in adipose and liver tissue of obese mice^46,47^. Genetic inactivation of *Ripk3* was found to increase Caspase-8-dependent adipocyte apoptosis and inflammation and lead to impaired glucose signaling in those cells^46^. However, this is the first report that connects necroptosis with diabetes in humans. Our evidence is based on ACMG guidelines for variant classification, which state that loss-of-function variants that segregate thoroughly with the phenotype, are considered pathogenic. The chances of this mutation occurring randomly with no connection to MODY are greatly diminished by the rarity of the mutation in the general population as well as its full segregation with the disease in the five affected family members.

The G316D MLKL mutation occurs in the C-terminal pseudokinase domain, where specific mutations in mouse MLKL have been found to either cause constitutive necroptosis or dampen it^16,48^, while missense mutations in the human domain reported so far, including this report, were shown to either abolish or delay the necroptotic function of the protein^17,24,36,39^. While dysregulated MLKL stability and activation has been observed in disease, MLKL is dispensable for animal development^16,25,26^, although human MLKL protein deficiency has been recently associated with neurodegenerative disease^26^. Previous studies have highlighted how prone mouse MLKL is to hyperactivation^16,27,37,49^ whilst mutations of the human counterpart typically suppress activation^39,42^. Consistent with these observations, exogenous expression of the patient MLKL substitution, G316D, in human cell lines led to 50–100% of the protein levels of the wild-type counterpart, whilst we did not observe evidence for decreased transcription in whole blood RNAseq analysis of our *MLKL*^*G316D/+*^ patients. These data do not indicate the transcript to be unstable and sensitive to nonsense mediated decay, nor the protein to be intrinsically unstable. Instead, the MLKL^G316D^ exhibited reduced capacity for activation by RIPK3 through phosphorylation at Ser358, which led to a corresponding abrogation of its necroptotic effector activity. Based on these data, it is predicted that cells from humans that are heterozygous for the MLKL^G316D^ mutation would exhibit diminished capacity for necroptosis. This reduction could be due to a simple monoallelic reduction of necroptosis-competent MLKL, but we cannot rule out the potential for dominant-negative impediment of MLKL^WT^ function by MLKL^G316D^ within cells. Because MLKL oligomerisation is an essential checkpoint in the execution of necroptotic cell death^37,39,42,44^, it is foreseeable that blockade of assembly of productive MLKL oligomers, such as by co-expression of an inactive mutant like MLKL^G316D^, could impose another layer of restriction on necroptotic signaling.

The choice of two human cell lines, HT29 and U937, to perform the above experiments was due to their known ability to reliably undergo necroptosis upon stimulation with defined stimuli^24,36,39,42,44^. A cellular system that would bring together the capacity to model PDX1-MODY and to study the MLKL’s necroptotic signaling functions in vitro, is not available currently. Similarly, there is currently no animal model for the study of human MLKL protein. As mentioned earlier, the activation and regulation of MLKL differ between human and mouse cells, and in particular, the pseudokinase domain (where the G316D mutation resides) has diverged between the two species. Thus, studying the effect of the mutation in the context of a PDX1-MODY animal model remains of outstanding interest for future studies.

While our studies clearly show the MLKL^G316D^ substitution to compromise MLKL function in necroptotic signaling, we cannot exclude other mutations and/or factors contributing to disease etiology in this family. Indeed, compelling cases can be made for further examination of the three other gene variants found in the affected but not unaffected siblings. Of particular interest is the finding that the variant in the ER stress protein, ERN1, is paternally, not maternally derived. Given the important role of the unfolded protein response in the etiology of diabetes (reviewed in Gosh et al.^50^), it is possible that this variant may have contributed to the earlier onset of diabetes in the affected siblings relative to their mother, who does not carry this ERN1 substitution. The evidence for a role of the magnesium ion transmembrane transporter, NIPAL1, in diabetes is scant; it was shown to positively influence glucose stimulated insulin secretion in pancreatic β-cell-like mouse cell line^51^. And SPTBN4, a spectrin that links cytoskeletal actin to the plasma membrane, does not yet have a known role in diabetes.

In summary, we have shown that the diabetic members of a non-penetrant PDX1-MODY family exclusively harbor a very rare damaging mutation in the necroptosis MLKL protein. Our in vitro assays confirm the deleterious effect of the mutation on MLKL activation via RIPK3-mediated phosphorylation and its subsequent necroptotic activity. We, therefore, postulate that compromised necroptosis contributes to diabetic disease pathogenesis by conferring full penetrance on the PDX1 mutation.

## Methods

### Sample collection and processing

Sample and data collection were performed under Sidra IRB protocol 1601002512 and the subjects consented to the research and publication of findings. For DNA analysis, saliva was collected in Oragene OG-500 tubes (DNA Genotek, Ottawa, Canada). DNA was isolated using QIAsymphony DSP DNA MIDI kit (Qiagen, Hilden, Germany), following the manufacturer’s recommendations. DNA quantity and quality were checked using a Nanodrop spectrophotometer (Thermo). DNA processed for whole genome sequencing was subjected to Illumina HiSeq sequencing, generating 150 bp paired end reads with 30x coverage. For RNA analysis, whole blood was collected in PAXgene Blood RNA tubes (BD Biosciences, San Jose, CA, United States). Blood was first centrifuged for 10 min at 5000 × *g* using a swing out rotor to collect the pellet. After decanting the supernatant, the pellet was resuspended in 300 μl of BR1 buffer by vortexing and processed on the QIAsymphony SP platform for automated extraction. To obtain mRNA libraries, polyA RNA selection is performed using an Oligo-dT magnetic bead system, followed by fragmentation and first strand synthesis using Superscript IV and second strand synthesis. The cDNA obtained after reverse transcription is ligated with TruSeq RNA Combinatorial Dual Index adapters and amplified for 15 cycles. cDNA was then sequenced on Illumina HiSeq4000 to an average of 18 million reads per sample.

### Genomic data processing

Genomic data were aligned to GRCh37 using bowtie^52^. SNPs and indels were called with GATK;^53^ CNVs were detected with NxClinical software v5.1 (BioDiscovery, Hawthorne, CA). Pathogenic variant analysis was done with Ingenuity Variant Analysis^TM^ software (QIAGEN, Inc.), NxClinical, and other open source tools. CNVs overlapping segments in the Database of Genomic Variants (DGV)^54^ or are common in our internal database of Middle Eastern subjects were excluded. We applied the following filters for SNPs indels: read depth >20, MAPQ > 30, base quality >20, Minor Allele Frequency (MAF) < 0.01 (1%), in silico prediction of pathogenicity by SIFT^55^ and PolyPhen^56^, and lack of common structural variation in the region (as per DGV).

### Cell lines, reagents and antibodies

*MLKL*^*−/−*^ HT29 human adenocarcinoma cell lines and *MLKL*^*−/−*^ U937 human monocytic cell lines were generated in house using CRISPR-Cas9 technology^39^. Recombinant hTNF was produced in-house and used at a final concentration of 100 ng/mL. Smac mimetic (Compound A), and the caspase inhibitor IDN-6556 were provided by TetraLogic (Malvern, PA, USA). Rat-anti hRIPK3 1H2 (1:1000)^24,57^ and rat anti-MLKL 3H1 (1:2000)^16^ (biotinylated and non-biotinylated forms) were produced in-house (3H1 available from Millipore as catalog number MABC604). Anti-human MLKL pS358 (1:1000, ab187091) and anti-Actin (1:10,000, ab5694) were purchased from Abcam. Horseradish peroxidase (HRP) conjugated goat anti-rat IgG (Southern Biotech 3010-05), HRP-conjugated goat anti-mouse IgG (Southern Biotech 1010-05), HRP-conjugated goat anti-rabbit IgG (Southern Biotech 4010-05) and HRP-conjugated streptavidin (Millipore SA202) were used for the secondary detection of primary antibodies (all 1:10,000).

### Expression constructs and cell culture

Genes encoding full length human MLKL (residues 1 to 471) were cloned into the lentiviral expression vector pFTRE3G PGK Puro^16^. Wild-type MLKL was encoded as before;^39^ the G316D substitution was introduced by oligonucleotide-directed PCR. The insert sequences in the arising constructs were verified by Sanger sequencing (AGRF, Melbourne). Cells were maintained at 37 °C, 10% CO_2_ in DMEM (HT29) or RPMI (U937) cell culture medium containing 8 % Fetal Calf serum and 2.5 μg/mL Puromycin as described previously^36,39^. Cell lines were routinely verified as free of mycoplasma contamination by PCR.

### Measuring cell death kinetics using quantitative live cell imaging

Both HT29 (adherent) and U937 (suspension) human cell lines were seeded at a density of 1.5 × 10^4^ cells/well in a 96 well plate and allowed to attach/settle for 48 h or 1 h respectively. Cells were stimulated as indicated in media containing Sytox Green (500 nM, ThermoFisher Scientific) and imaged at 1 h intervals using an IncuCyte S3 Live cell imager.

Numbers of Sytox positive cells, percentage confluence (for HT29) and total cell number (for U937) were quantified using IncuCyte measurement of green fluorescent and phase contrast signals and IncuCyte image analysis software. HT29s cultures were inspected for similar confluence and cell death quantified as number of Sytox Green positive cells/mm^2^. U937 cultures were assessed as number of Sytox Green positive cells/mm^2^ as a proportion of total phase contrast objects /mm^2^.

### Measuring protein levels in cells

HT29s were seeded at a density of 4 × 10^5^ cells/well in a 24 well plate and allowed to attach for 48 h. U937s were seeded at a density of 4.5 × 10^5^ cells/well in a 48 well plate and allowed to settle for 1 h. Doxycycline (100 ng/mL), TNF (100 ng/mL), Smac mimetic (500 nM) and IDN-6556 (5 μM) were added simultaneously. Cells were collected after 5 h and directly boiled in reducing sample loading buffer for separation by SDS-PAGE and western blotting.

### Statistics (cell death assays)

All cell death data are plotted as mean ± SEM with number of independent experiments (n) indicated in figures. Independent experimental repeats (“n”) include both biological repeats (independently generated *MLKL*^*−/−*^ cell lines used on the same day) and experimental repeats (same cell lines used in experiments repeated on different days).

## Supplementary information

Supplementary Figure 1

Supplementary Figure 2

Supplementary Figure 3

## Data Availability

The datasets generated during and/or analyzed during the current study are not publicly available due to privacy protection but are available from the corresponding author upon request.

## References

[1] American Diabetes A. 2. (2018). Classification and diagnosis of diabetes: standards of medical care in diabetes-2018. Diabetes Care.

[2] Patel KA (2017). Heterozygous RFX6 protein truncating variants are associated with MODY with reduced penetrance. Nat. Commun..

[3] Macfarlane WM (1999). Missense mutations in the insulin promoter factor-1 gene predispose to type 2 diabetes. J. Clin. Invest.

[4] Gragnoli C (2005). IPF-1/MODY4 gene missense mutation in an Italian family with type 2 and gestational diabetes. Metabolism.

[5] Stoffers DA, Ferrer J, Clarke WL, Habener JF (1997). Early-onset type-II diabetes mellitus (MODY4) linked to IPF1. Nat. Genet.

[6] Hani EH (1999). Defective mutations in the insulin promoter factor-1 (IPF-1) gene in late-onset type 2 diabetes mellitus. J. Clin. Invest.

[7] Weng J (2001). Functional consequences of mutations in the MODY4 gene (IPF1) and coexistence with MODY3 mutations. Diabetologia.

[8] Chen C, Sibley E (2012). Expression profiling identifies novel gene targets and functions for Pdx1 in the duodenum of mature mice. Am. J. Physiol. Gastrointest. Liver Physiol..

[9] De Franco E (2013). Biallelic PDX1 (insulin promoter factor 1) mutations causing neonatal diabetes without exocrine pancreatic insufficiency. Diabet. Med.

[10] Hashimoto H (2015). Expression of pancreatic and duodenal homeobox1 (PDX1) protein in the interior and exterior regions of the intestine, revealed by development and analysis of Pdx1 knockout mice. Lab Anim. Res.

[11] Brissova M (2002). Reduction in pancreatic transcription factor PDX-1 impairs glucose-stimulated insulin secretion. J. Biol. Chem..

[12] Trojanowski B (2020). Elevated beta-cell stress levels promote severe diabetes development in mice with MODY4. J. Endocrinol.

[13] Karczewski KJ (2020). The mutational constraint spectrum quantified from variation in 141,456 humans. Nature.

[14] Landrum MJ (2018). ClinVar: improving access to variant interpretations and supporting evidence. Nucleic Acids Res.

[15] Murphy JM (2020). The killer pseudokinase mixed lineage kinase domain-like protein (MLKL). Cold Spring Harb. Perspect. Biol.

[16] Murphy JM (2013). The pseudokinase MLKL mediates necroptosis via a molecular switch mechanism. Immunity.

[17] Sun L (2012). Mixed lineage kinase domain-like protein mediates necrosis signaling downstream of RIP3 kinase. Cell.

[18] Zhao J (2012). Mixed lineage kinase domain-like is a key receptor interacting protein 3 downstream component of TNF-induced necrosis. Proc. Natl Acad. Sci. USA.

[19] Guo H (2015). Herpes simplex virus suppresses necroptosis in human cells. Cell Host Microbe.

[20] Kaiser WJ, Upton JW, Mocarski ES (2013). Viral modulation of programmed necrosis. Curr. Opin. Virol..

[21] Nailwal, H. & Chan, F. K. Necroptosis in anti-viral inflammation. *Cell Death Differ*. 4–13 (2019). 10.1038/s41418-018-0172-x PMC6294789 30050058

[22] Pearson JS (2017). EspL is a bacterial cysteine protease effector that cleaves RHIM proteins to block necroptosis and inflammation. Nat. Microbiol.

[23] Pearson JS, Murphy JM (2017). Down the rabbit hole: Is necroptosis truly an innate response to infection?. Cell Microbiol.

[24] Petrie EJ (2019). Viral MLKL homologs subvert necroptotic cell death by sequestering cellular RIPK3. Cell Rep..

[25] Wu J (2013). Mlkl knockout mice demonstrate the indispensable role of Mlkl in necroptosis. Cell Res..

[26] Faergeman SL (2020). A novel neurodegenerative spectrum disorder in patients with MLKL deficiency. Cell Death Dis..

[27] Hildebrand JM (2020). A missense mutation in the MLKL brace region promotes lethal neonatal inflammation and hematopoietic dysfunction. Nat. Commun..

[28] Rickard JA (2014). TNFR1-dependent cell death drives inflammation in Sharpin-deficient mice. Elife.

[29] Dannappel M (2014). RIPK1 maintains epithelial homeostasis by inhibiting apoptosis and necroptosis. Nature.

[30] Anderton H, Rickard JA, Varigos GA, Lalaoui N, Silke J (2017). Inhibitor of apoptosis proteins (IAPs) limit RIPK1-mediated skin inflammation. J. Invest Dermatol.

[31] Wang B (2018). A rare variant in MLKL confers susceptibility to ApoE varepsilon4-negative Alzheimer’s disease in Hong Kong Chinese population. Neurobiol. Aging.

[32] Muller T (2017). Necroptosis and ferroptosis are alternative cell death pathways that operate in acute kidney failure. Cell Mol. Life Sci..

[33] Cho YS (2009). Phosphorylation-driven assembly of the RIP1-RIP3 complex regulates programmed necrosis and virus-induced inflammation. Cell.

[34] Cook WD (2014). RIPK1- and RIPK3-induced cell death mode is determined by target availability. Cell Death Differ..

[35] He S (2009). Receptor interacting protein kinase-3 determines cellular necrotic response to TNF-alpha. Cell.

[36] Davies KA (2020). Distinct pseudokinase domain conformations underlie divergent activation mechanisms among vertebrate MLKL orthologues. Nat. Commun..

[37] Hildebrand JM (2014). Activation of the pseudokinase MLKL unleashes the four-helix bundle domain to induce membrane localization and necroptotic cell death. Proc. Natl Acad. Sci. USA.

[38] Murphy JM (2014). Insights into the evolution of divergent nucleotide-binding mechanisms among pseudokinases revealed by crystal structures of human and mouse MLKL. Biochemical J..

[39] Petrie EJ (2018). Conformational switching of the pseudokinase domain promotes human MLKL tetramerization and cell death by necroptosis. Nat. Commun..

[40] Petrie EJ, Czabotar PE, Murphy JM (2019). The Structural Basis of Necroptotic Cell Death Signaling. Trends Biochem Sci..

[41] Garnish, S. E. et al. Conformational interconversion of MLKL and disengagement from RIPK3 precede cell death by necroptosis. *Nat Commun.*10.1038/s41467-021-22400-z (2021). 10.1038/s41467-021-22400-z PMC8044208 33850121

[42] Petrie EJ (2020). Identification of MLKL membrane translocation as a checkpoint in necroptotic cell death using Monobodies. Proc. Natl Acad. Sci. USA.

[43] Samson AL, Garnish SE, Hildebrand JM, Murphy JM (2021). Location, location, location: a compartmentalized view of necroptotic signaling. Sci. Signal.

[44] Samson AL (2020). MLKL trafficking and accumulation at the plasma membrane control the kinetics and threshold for necroptosis. Nat. Commun..

[45] Wang H (2014). Mixed Lineage Kinase Domain-like Protein MLKL Causes Necrotic Membrane Disruption upon Phosphorylation by RIP3. Mol. Cell.

[46] Gautheron J (2016). The necroptosis-inducing kinase RIPK3 dampens adipose tissue inflammation and glucose intolerance. Nat. Commun..

[47] Xu H (2019). The pseudokinase MLKL regulates hepatic insulin sensitivity independently of inflammation. Mol. Metab..

[48] Tanzer MC (2015). Necroptosis signalling is tuned by phosphorylation of MLKL residues outside the pseudokinase domain activation loop. Biochemical J..

[49] Jacobsen AV (2016). HSP90 activity is required for MLKL oligomerisation and membrane translocation and the induction of necroptotic cell death. Cell Death Dis..

[50] Ghosh R, Colon-Negron K, Papa FR (2019). Endoplasmic reticulum stress, degeneration of pancreatic islet beta-cells, and therapeutic modulation of the unfolded protein response in diabetes. Mol. Metab..

[51] Manialawy Y., Khan S. R., Bhattacharjee A., Wheeler M. B. The magnesium transporter NIPAL1 is a pancreatic islet-expressed protein that conditionally impacts insulin secretion. *J. Biol. Chem.* 2020: jbc. RA120. 013277. 10.1074/jbc.RA120.013277 PMC7380176 32439805

[52] Langmead B, Trapnell C, Pop M, Salzberg SL (2009). Ultrafast and memory-efficient alignment of short DNA sequences to the human genome. Genome Biol..

[53] Van der Auwera GA (2013). From FastQ data to high‐confidence variant calls: the genome analysis toolkit best practices pipeline. Curr. Protoc. Bioinforma..

[54] MacDonald JR, Ziman R, Yuen RK, Feuk L, Scherer SW (2014). The Database of Genomic Variants: a curated collection of structural variation in the human genome. Nucleic Acids Res..

[55] Vaser R, Adusumalli S, Leng SN, Sikic M, Ng PC (2016). SIFT missense predictions for genomes. Nat. Protoc..

[56] Adzhubei IA (2010). A method and server for predicting damaging missense mutations. Nat. Methods.

[57] Samson A. L., et al. A toolbox for imaging RIPK1, RIPK3, and MLKL in mouse and human cells. Cell Death Differ. 10.1038/s41418-021-00742-x (2021) 15 Feb 2021. 10.1038/s41418-021-00742-x PMC8257593 33589776

[58] Venselaar H, Te Beek TA, Kuipers RK, Hekkelman ML, Vriend G (2010). Protein structure analysis of mutations causing inheritable diseases. An e-Science approach with life scientist friendly interfaces. BMC Bioinforma.

