# Supplementary figures and images for: A family harboring an MLKL loss of function variant implicates impaired necroptosis in diabetes
Source: Cell Death Dis. 2021 Apr 1;12(4):345. doi: 10.1038/s41419-021-03636-5 (PMC8016849; doi:10.1038/s41419-021-03636-5)

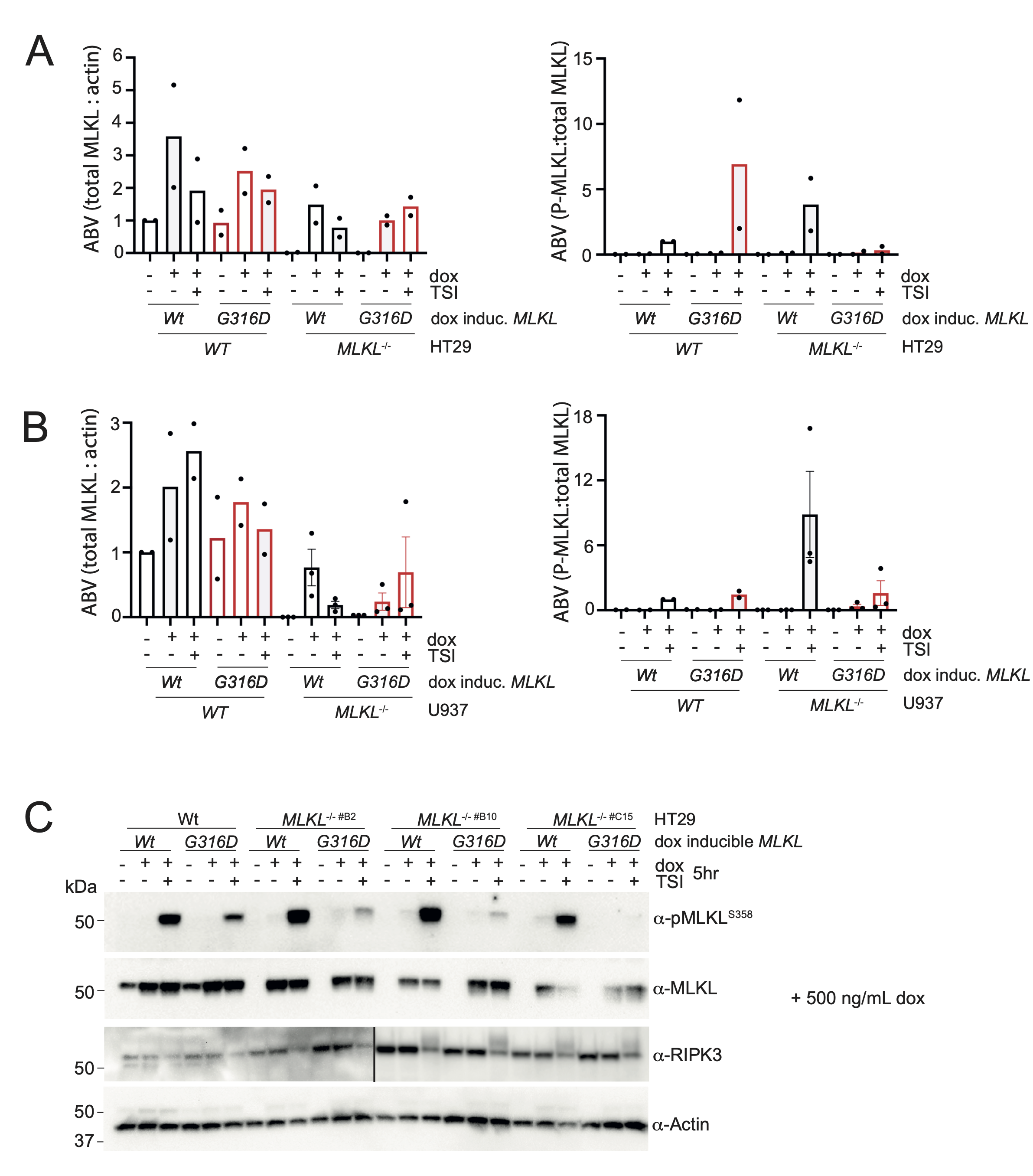

Supplement: Supplementary file 1 — Supplementary Figure 1 [file 41419_2021_3636_MOESM1_ESM.png]

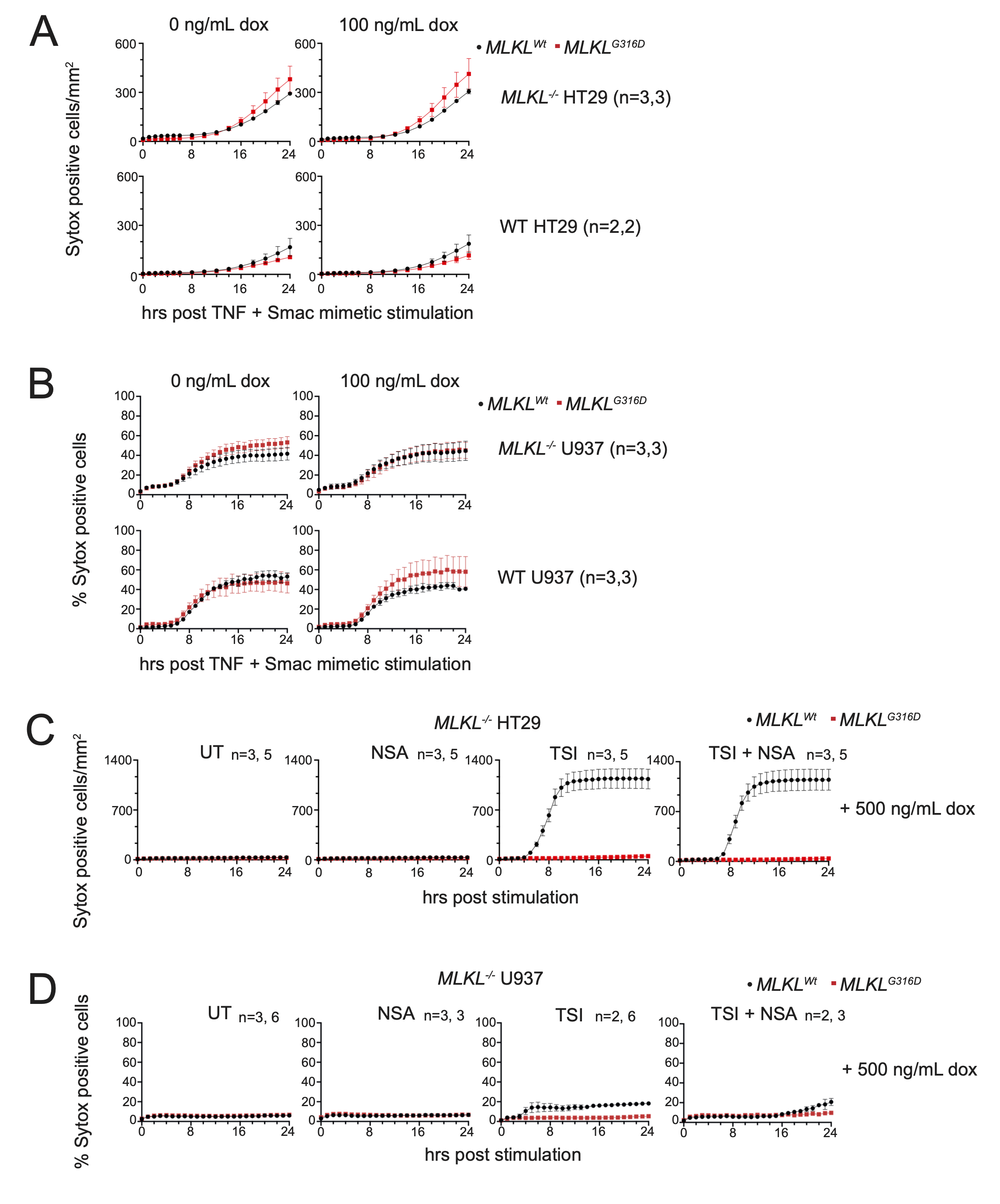

Supplement: Supplementary file 2 — Supplementary Figure 2 [file 41419_2021_3636_MOESM2_ESM.png]

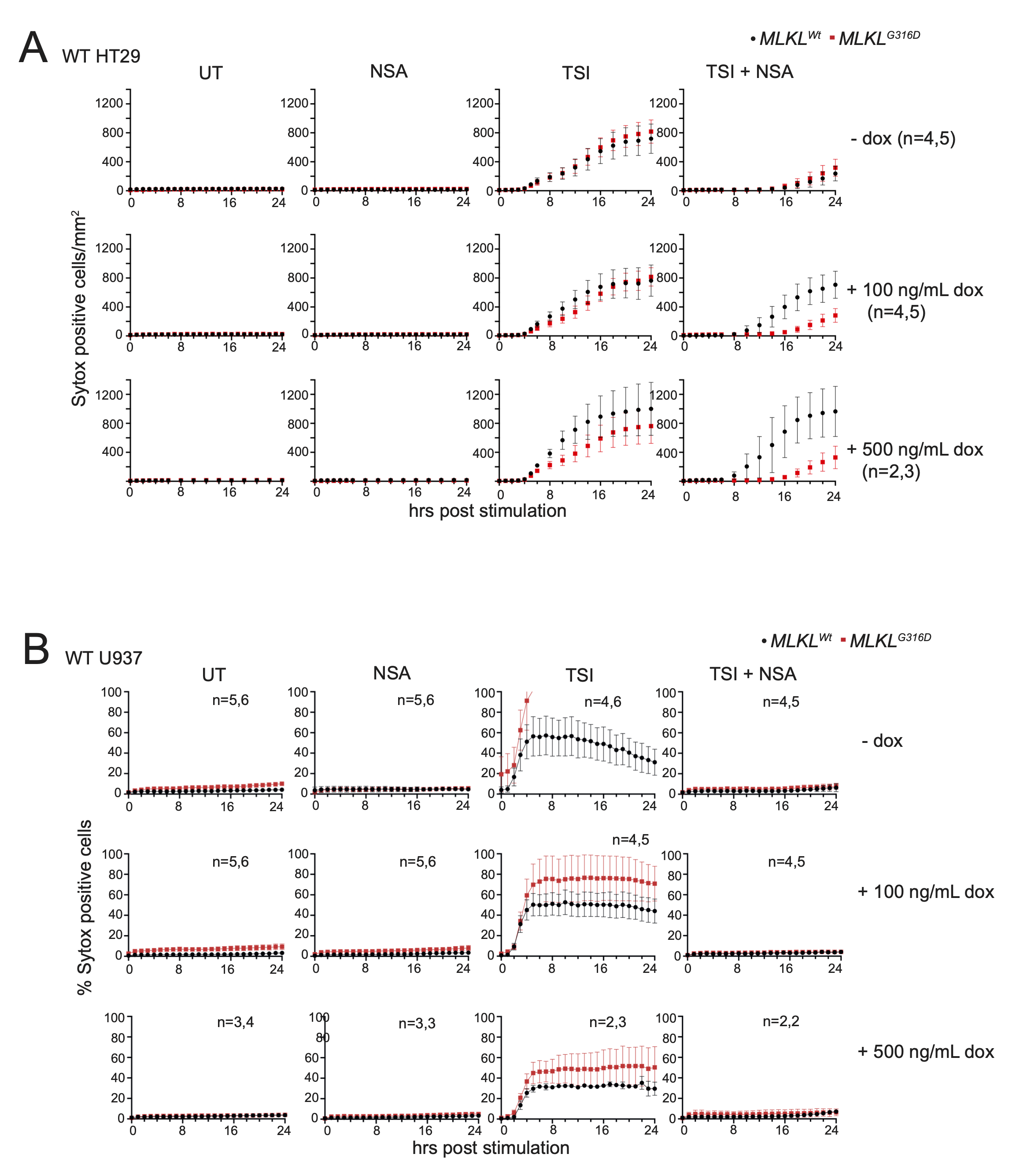

Supplement: Supplementary file 3 — Supplementary Figure 3 [file 41419_2021_3636_MOESM3_ESM.png]
